# Clinical oncology in resource-limited settings

**DOI:** 10.1186/1750-9378-8-39

**Published:** 2013-10-07

**Authors:** Franco M Buonaguro, Serigne N Gueye, Henry R Wabinga, Twalib A Ngoma, Jan B Vermorken, Sam M Mbulaiteye

**Affiliations:** 1 Division of Molecular Biology & Viral Oncology, Department of Experimental Oncology, Istituto Nazionale Tumori -IRCCS “Fond Pascale”, Naples, Italy; 2 Division of Urology and Andrology, Grand Yoff General Hospital – Department of Surgery/Urology, University Cheikh Anta DIOP, Dakar, Senegal; 3 Department of Pathology, College of Health Sciences, Makerere University, Kampala, Uganda; 4 Ocean Road Cancer Institute, Dar es Salaam, Tanzania; 5 Department of Medical Oncology, Antwerp University Hospital, Antwerp, Belgium; 6 Infections and Immunoepidemiology Branch, Div of Cancer Epidemiology and Genetics, National Cancer Institute, National Institutes of Health, Department of Health and Human Services, 9609 Medical Center Dr, Rm. 6E118 MSC 9704, Bethesda, MD 20892-9704, USA

## Abstract

*Infectious Agents and Cancer* is introducing a new section of Clinical Oncology with the main objective of stimulating debate through articles published in the section. Infectious diseases have been the major causes of morbidity and mortality in human populations, and have dominated the medical approach to clinical and public health. Successful efforts to control mortality from acute infections have paved the way for chronic, mostly indolent, infections to become major causes of morbidity. Cancer, hitherto thought to be rare in resource-limited settings, is becoming a major contributor. The changes in mortality patterns are due, in part, to diseases linked to rapid changes in lifestyle, urbanization, and pollution. These diseases include many of the non-infection associated cancers. However, there is a dearth of information about the burden, pathogenesis, and therapeutic approaches about cancer in resource-limited countries. There are also substantial other challenges, including economic, infrastructure, technology, and personnel. The *Journal* advocates for interactive local–global (lo-bal) efforts to generate relevant knowledge about cancer burden, pathogenesis, and therapeutic approaches using a bottom-up approach to sharpen the focus on local and global relevance of research and clinical and public practice, particularly in resource-limited countries. The section on Clinical Oncology in *Infectious Agents and Cancer* will harness these “lo-bal” strategies to reduce substantially the time from concept, discovery, and development and implementation of locally and globally applicable diagnostic and therapeutic technologies.

## Editorial

Do we really need a clinical oncology journal focusing on resource-limited countries? Many, perhaps, would be inclined to answer “no”. Resource-limited countries are faced with extraordinary burdens of acute and chronic infectious agents, malnutrition, civil disturbance, violence, and economic disparity and have to make, often, painful choices how to spend their limited resources. Intuitively, those countries should focus on identifying diseases that rank top in their morbidity surveys and learning and applying strategies for early diagnosis and treatment of those conditions. Because previous morbidity surveys have indicated that acute infections, malnutrition, and maternal conditions are the leading causes of morbidity and mortality, the argument to establish and develop clinical and basic oncology services in resource-limited countries seems weak.

Yet, morbidity and mortality from cancer is substantial in many resource-limited countries. The successful efforts to control mortality from acute infections have paved the way for chronic infections to become the major causes of morbidity in resource-limited settings. Inevitably, cancer will also become important. Infection-associated cancers already contribute to more than one-third of cancers in resource-limited settings, compared to less than one-fifth in developed countries. Concomitant increases in mortality from other diseases, which are linked to rapid changes in lifestyle, urbanization, and pollution, will undermine the gains from decreased mortality from acute infections. Clinicians working in resource-limited countries regularly encounter patients with cancer, many patients present with late symptoms. Clinicians confront the challenges to diagnose, treat and rehabilitate patients with cancer. Public health managers confront the challenges to balance resource allocation between cancer services, for which the demand is growing steeply, and the need to meet priorities imposed by acute infections, malnutrition, and maternal disease for which the demand has historically been high.

We lack robust data from well-designed and well-conducted studies in resource-limited settings, which complicates forecasting. Likewise, there is a dearth of information about pathogenesis, metabolic or genetic pathways and therapeutic approaches, which hampers efforts towards rational programs to mitigate the cancer burden in resource-limited countries. The challenges for developing evidence-based oncologic programs in resource-limited settings are broad and diverse.

However, there are many reasons to develop robust oncologic programs in resource-limited countries. These include humanitarian considerations: the need to provide a basic diagnosis, treatment or pain care and rehabilitation. Others include scientific considerations to estimate the burden, distribution, and trends of cancer and conduct in-depth studies of the biology of cancer. Basic science studies are necessary to develop targeted therapies. The increasing appreciation of the substantial contribution of chronic infections to cancer provides strong motivation to identify infection-associated cancers and to deploy infection control strategies to cancer control.

Infections have historically exerted tremendous evolutionary pressure on human populations. For example, pressure from malaria infections has left the glucose-6-phosphate dehydrogenase (G6PD) signature mutation at nucleotide 563 (Exon 6). This mutation leads to a substitution of a phenylalanine amino acid with a serine amino acid at position 188 (SER188PHE) and it is associated with G6PD deficiency anemia and reduced risk of death from malaria [1], as initially proposed by Davidson et al., 1964 [2]. Similarly, other severe fatal infections, such as plague, smallpox HIV infection, have induced signature mutations, including the CCR5 deletion (i.e. CCR5 Δ32), which is associated with resistance to these diseases [3], but it might also increase susceptibility to other infections, such as the West Nile infections [4]. Other infections show peculiar distributions, but whether these disparities may be associated with signature mutations and/or specific concurrent infections or other local environmetal co-factors is unknown. For example, human herpesvirus 8 (HHV-8), the viral cause of Kaposi’s sarcoma (KS), shows a peculiar gradient that mirrors KS incidence [5], but genetic factors for this disease as well as other cofactors, able to explain the different susceptibility to the infection, are largely unknown. Another important virus that shows disparity is hepatitis C virus (HCV) whose prevalence in Europe is <2.5%, but is extremely high in some populations including in Southern Italy where the prevalence in people >65 years of age is >30%. The incidence of hepatocellular carcinoma incidence tracks imperfectly with HCV prevalence [6,7], but whether genetic factors or local environmental co-factors contribute is unknown. Finally, the higher incidence of colon cancer could be associated with meat consumption, as recently raised by Nobel laureate Harold zur Hausen [8]. Could the habit of eating raw meat be a factor in transmitting infections from animals to humans? The Euro-Asiatic bovines (Bos taurus) and Indian Zebu (Bos indicus) or their hybrids Bos taurus Africanus (such as Sanga or the Ugandan race Ankole-Watusi) are less susceptible to bovine infections, such as rinderpest, thus favoring them over other bovine types. Is it possible that these cows also transmit other pathogens to humans that increase the risk of cancers? Studies conducted in resource-limited settings could be informative.

While serendipitous findings, such as the discovery and identification of HPV16 in cervical cancer from African patients [9] and of several HPV18 copies in HeLa cervical cancer cells [10], have driven science in the past, such opportunities arise only in the context of ongoing sustained research activity. For example, observation of extraordinarily high incidence of penile cancers in Ugandan tribes, where penile cancer represented >40% of all male cancers was observed when research was introduced in Africa [11,12], and suggested effects of local factors. However, when civil disturbance disrupted nascent research efforts in Africa, no further scientific studies were appropriately performed and the underlying local risk factors for cancer were not identified. More recently, following the HIV epidemic, a mini-epidemic of conjunctival cancers has been reported in Africa [13], but not in the West. While the descriptive data may not lead to definitive scientific interpretation, integration of molecular data could substantially increase their relevance. Taken together, study of cancer in resource-limited settings has relevance to local communities as well as to the international community through knowledge generation [14].

For these reasons, *Infectious Agents and Cancer* is introducing a new section on Clinical Oncology to stimulate debate in the scientific community, along with the pharmaceutical companies, to act in concert to support nascent oncologic programs in resource-limited settings. This debate will lead to identification of the cheapest available route to a complete and effective anti-cancer regimen, for it to be pursued and implemented as for acute infectious diseases [15]. This approach will help meet the needs in limited-settings, and also bring mutual benefits to communities in wealthy countries. In a global village no one component can be left out. Wholesome clinical experience must be shared; the technological advances must be exchanged. This bottom-up approach may be referred to as the “lo-bal” strategy, where local observations are analyzed with scientific methods and the results are extrapolated for global comprehension of the phenomenon. Innovative pathogenesis studies in African populations may lead to discovery of novel genetic pathways or targets for new therapeutic approaches [16]. An integration of technological advanced oncologists with the local bio-medical community will accelerate the general scientific knowledge, while optimizing local public health programs and the general health conditions.

**Figure 1:**
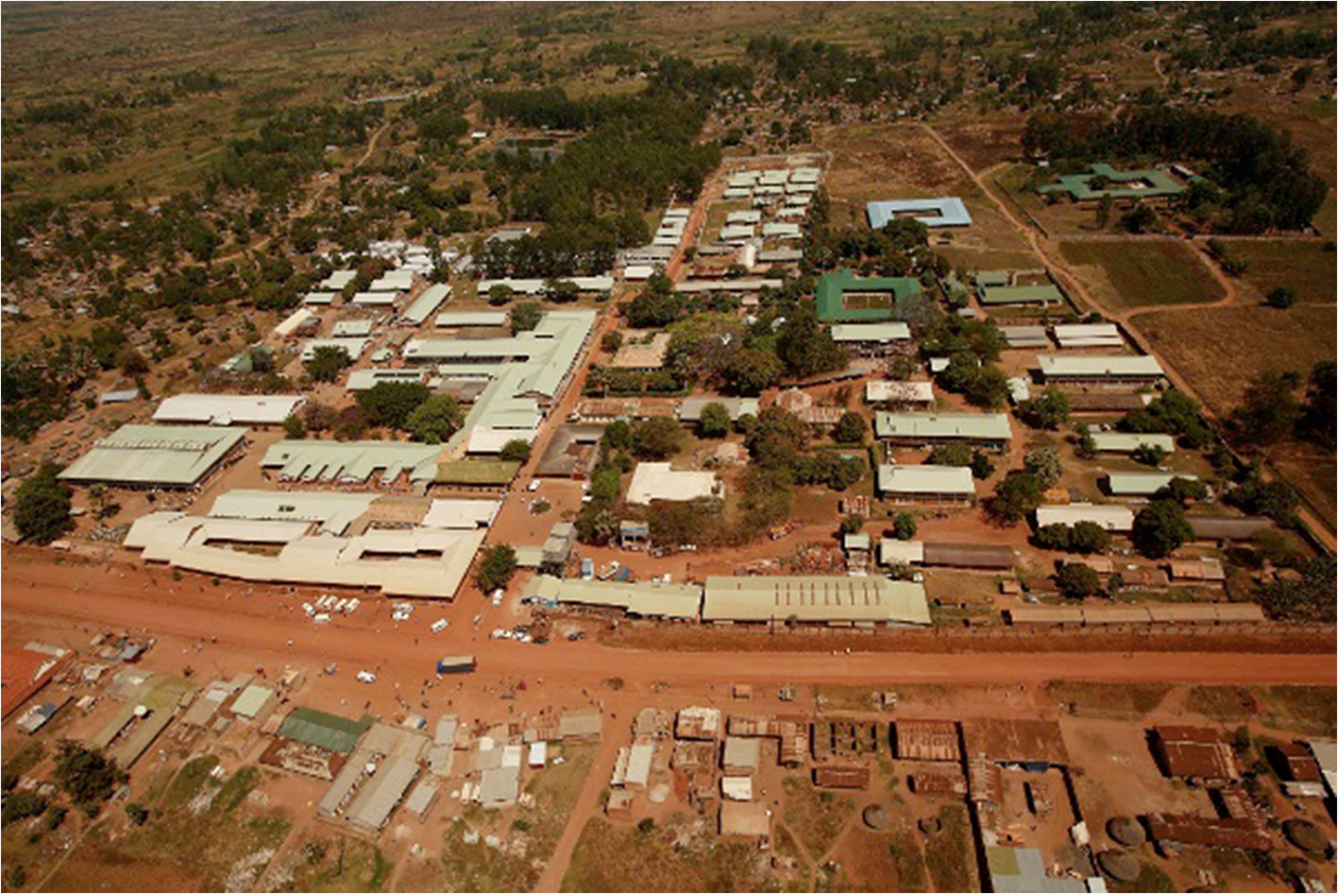
The St Mary’s Hospital at Lacor, Gulu - Uganda.

Young doctors from newly established Medical school lost in the bush of the dark Africa, such as at St. Mary’s Hospital, Lacor, in Uganda, (Figure 1) have applied themselves to medical science. In one example, they showed that some protocols developed outside Africa, may not be easily or usefully translated to the local conditions, highlighting the need for better interaction between the more advanced scientific community and local teams [17,18]. The FEAST trial project, which won the BMJ Research Paper of the Year 2012 [19], is an excellent example of this. In the same hospital (Figure 1) local clinicians and nurses were able to identify and mount rapid containment, albeit at some cost of their lives, an Ebola epidemic and “produce more scientific data on Ebola”. These examples indicate a thirst to learn and practice that good medicine, even extreme in terms of economic conditions and constructive interaction with the global scientific community [20,21].

The “lo-bal” strategy is well developed for acute infectious diseases. It will definitely foster the sharing of experiences and information between worldwide oncologist as well as the identification and development of diagnostic and therapeutic protocols globally valid in the current global village. *Infectious Agents and Cancer* hopes to stimulate and contribute to this debate through articles published in the section of Clinical Oncology.
